# Compression of the Aorta as a Means of Arresting Uterine Hemorrhage

**Published:** 1841-09

**Authors:** 


*Compression of the Aorta as a means of arresting Uterine Hemorrhage.*—Dr. Piedagnel communicated to the *Societe Medicale d'Emulation*, on the 4th of April, 1840, two cases in which uterine hemorrhage, following labour, was immediately arrested by compression of the aorta. These cases were referred to MM. Velpeau and Brierre de Boisment, who made a report in relation to them on the 6th of May. This last paper contains so admirable a summary of the history of this therapeutic measure, that we are sure we shall gratify and instruct our readers by transferring the principal part of it to our pages.

"The idea of compressing the aorta to arrest uterine hemorrhage, must naturally have suggested itself as soon as the laws of anastomsis became well known, and accoucheurs saw the splendid conquests of modern surgical art in the vast field of aneurisms. But before investigating the origin of hemorrhages and discussing the value of compression, it is not without utility, in a few words, to trace the history of this new mode of cure. When MM. Trehan and Baudeloque (nephew) published their memoirs,* compression had not been practised

---

*Trehan, p. 1. Nouveau traitement des hemorrhagies uterines qui suivent l'accouchement par la compression de l'aorte.—Paris, 1829; in 8vo., p. 29. Baudeloque neveu, Traitement des pertes de sang qui peuvent suivre l'accouchement, par la compression do l'aorte exercee sur le ventre, la pression convenable du ventre et l'usage du seigle ergote et des fortifians.—*Journ. des Conn. Med. Ch.*, 1834, t. i., p. 201.

at all in France, but the moment that attention had been called to this subject, the ancients were searched, and foreign journals were hunted through; and soon, according to invariable custom, it was found that many authors before MM. Trehan and Baudeloque had spoken of this hemostatic remedy. This time we cannot go back to Hippocrates, and the honors of this priority fall to Daniel Louis Budiger, an accoucheur of Tubingen. In order to ascertain the real truth of these assertions, we have consulted the works referred to; and here is the result of these researches: Budiger employed compression of the aorta for the first time in a female who had just laid in and was at the point of death from loss of blood. The flooding lessened immediately; the uterus contracted; the mother was revived and saved. Budiger adds that he has had recourse to this remedy in a score of cases.

"Ploucquet published the new operation, the description of which had appeared in the Journal of Loder in 1797. He says positively that compression of the aorta had been made *per manum in utero adhuc expanso*.*

"Compression of the aorta to arrest uterine hemorrhages had been proposed, or at least employed nearly at the same epoch, by the celebrated Danish accoucheur, Math. Saxtorph. He compressed this vessel by acting on the womb through the walls of the abdomen.†

"In 1812 Boer expressed himself thus: 'Inter alia conamina nuper etiam hoc legimus; inductâ manu posterior uteri paries satis opprimitur ut, descendentis retro aortæ, velut suffocatione sanguis irruere in interum per subditos ramos præpediatur. Ecce nova alia procul ægris excogitata instructio! Quam circa candidus afferans quæ duobus periculis sum expertus. Ubi nempè uterus modice crassus et contractus est, compressio arteriæ, etiam si fieri possit, inefficax, ac ne quidem necessaria est. At flaccido et amplo viscere, ut manus robur in arteriam penetret, mors alioqui fores pulsat, ex uteri paresi scilicet, cujus hemorrhagia solum consequentia est, ut, nisi apoplexiam loci sustuleris, ægra occidat, sanguis fluat, necne. Id saltem compertum in præsens ego habeo."‡ This

*Loder's Journal, fur die Chirurgic Geburtshulfe und Gerichtliche Arzneikunde, b. 1, p. 493, 1797; vide t. ii of Ploucquet, Repert. Medicinæ Practicæ Chirurgiæ atque rei Obstetricæ, art. *Hemorrhagia, Compressio aortæ descendentis*, p. 261. Tubingæ, 1808.

†Math. Saxtorph's Gesammelete Schriften Geburtshulflichen, praktischen und physiologischen inhalts. Complete works of Math. Saxtorph, published by P. Scheil, Copenhagen, p. 229.—1803.

‡Boer, Nat. Med. Obstet. lib. sept., p. 525.—Vienna, 1812.

passage from Boer's work conclusively establishes that compression of the aorta was well known in Germany; that accoucheur, however, entertains an unfavorable opinion of this procedure, although it is probable that he objects to the manner of doing it. At the present day compression in the uterus is generally abandoned.

"Dr. Ulsamer, of Wurtzbourg, repeatedly compressed the aorta with success. The cases cited by him in his work are clear and precise.* He used two fingers to arrest the course of the blood. Nearly at the same period Eichelberg, Siebold, MM. Baudeloque, nephew, and Trehan reported cases of cure of uterine flooding by compression of the aorta. The two latter physicians, who were not aware of the labours of the Germans, believed themselves to be the inventors of this operation, and laid claim to the priority in it. Siebold's researches were made in 1828. In the case cited by him the aorta was compressed with the fist.

"We shall not dwell longer on this order of facts; they put it beyond a doubt that the treatment of uterine floodings by compression of the aorta is by no means new, and if honorable physicians have insisted on their having discovered this expedient, it is because, at the epoch of their proclaiming it, the works of the Germans were very little known, owing to the difficulties presented by their language.

"Compression of the aorta is then unquestionably registered in the annals of our science; but has this remedy all the efficacy attributed to it, or are we to believe, with some that it is useless and even hurtful? Two opinions so opposite can only arise from the mode of explaining the orgin of uterine hemorrhage.

"If the blood be furnished by the arteries—and the anatomcal disposition of the vessels, which acquire a prodigious development, favors this opinion—the theory would teach us to compress the main trunk; but if the blood comes from the venous system, as M. Jacquemier assures us it does, compression of the aorta would have none of the utility accorded to it. The last named author, founding his opinion on an examination of the vascular circle of the uterus, and of the manner in which the venous circulation is effected there, ascribes flooding to a want of resistance of the utero-placental veins. Whatever tends to favor the stasis of blood in the uterine veins a little too long, establishes a predisposition to repeated

---

See Friederich et Hesselbach: Beitraege zur Natur und Heilkunde, t i, p. 261.—1825.

losses. The quantity of blood furnished by the utero-placental arteries must be infinitely small.

"Compression of the aorta, continues Dr. Jacquemier, cannot attain the end proposed. By that measure we force the blood to pass more rapidly and abundantly through the division of the aorta above the point compressed; the upper vena cava bringing to the right auricle more blood than usual, the lower vena cava remains in a state of distension. The ovarian and uterine veins destitute of valves, partaking of the overloaded state of the inferior vena cava, it follows that the blood retrogrades into the uterine cavity, as long as the contraction of the uterus does not serve as a valve or check."*

"It cannot be denied that M. Jacquimier's explanation is ingenious; but we must admit that the mechanism of uterine floodings is similar to that of other hemorrhages; if it be so, the capillaries ought to have a large share in the production of these hemorrhages, which take place then by a true exhalation from the internal surface of the uterus. This explanation, favorable to compression of the aorta, is moreover justified by the facts already cited, and by the observations we are about to report.

"But if compression of the aorta be, as M. Velpeau thinks, a resource at once important and easy, it must not be lost sight of, that in thus suspending the afflux of arterial blood, we may equally check the return of venous blood, and that we ought, as much as possible, to avoid, at the same time, compressing the vena cava.

"M. Piedagnel's observations are two in number; the first was the case of a young lady, whose confinement, which had been natural, was followed by a flooding which cold water injections could not arrest. This physician then had recourse to compression of the aorta, seconded by the employment of refrigerants. The patient presented most of the symptoms observed in persons perishing of hemorrhage. As soon as he had put this expedient in operation, the flow ceased, but the convalescence was long.

"Observation second relates to the same lady. Her flooding was immediately combated with compression of the aorta; the blood stopped running, but the womb remained inert; a stream of cold water, directed into the interior of the organ,

---

*Recherches d'anatomie, de physiologie et de pathologie sur l'uturus humain pendant la gestation, et sur l'apoplexie utero-placentaire, pour servir a l'histoire des hemorrhagies uterines, du part premature et abortif; par M. Jacquemier.—*Arch. Gen. de. Med.*, 1839.

brought on contraction. Napkins placed above and maintained by a body bandage completed the cure. The duration of the flow, in this instance, was from 20 to 25 minutes.

"There is no accoucheur who, in the course of his practice, has not seen women recently delivered losing blood enough to bring on swooning, dimness of sight, and partial fainting, and these symptoms spontaneously disappearing at the moment we are about having recourse to energetic treatment. At other times cold applications and astringent drinks have sufficed to suspend the discharge of blood. In many cases we have availed ourselves most successfully of stimulation of the uterus, whose contractions we have excited by passing the ends of the fingers over the internal surface of the organ, while we compressed the womb with the left hand on the abdomen. Finally, the administration of ergot has very often triumphed over uterine hemorrhages. Perhaps the employment of some one of these means would have sufficed in the second case; but M. Piedagnel, who still had in his mind the grave symptoms of the former hemorrhage, acted prudently in resorting to the remedy which had succeded so well with him the first time.

"The interest attached to this subject engages us to report two other cases, which we owe to the kindness of our honorable confrère, M. Pinel Grandchamp. A lady, after a laborious delivery, in 1834, was taken with a considerable flooding. M. Pinel, time after time, introduced his hand into the vagina and uterus, to extract the clots and excite the internal face and neck of this viscus, expressed into the womb the juice of several lemons, and made free aspersions of cold water, all in vain, the swoons rapidly succeeded one another. In this state of things our colleague thought of compression of the aorta, and practised it for an hour. When he intercepted fully the passage of the blood in the artery, he observed the following phenomena:

"The countenance regained a portion of its natural color; the eyes became more animated, the lips more rosy; the pulse rose again; it was frequent, and had some fulness; the strength of the heart was increased; the patient came out of the state of syncope or prostration, into which a slight compression had almost immediately thrown her. She then said that she felt much better. The blood no longer flowed externally, although the uterus and vagina were freed from clots.

"When he suspended the compression, the blood no longer issued as abundantly, or with the same force, but if it recommenced flowing, all the phenomena of syncope reappeared, and the pulse almost ceased beating. Only an hour and a half after the operation, the uterus began to recover itself and contract so as no longer to create any fear of inertia. Twelve days afterwards the patient was perfectly restored.* No ergot whatever was administered; it would probably, adds M. Pinel, have aided me in more quickly overcoming the inertia of the uterus.

"In the course of that year M. Pinel Grandchamp was called, in consultation, by Dr. Marye, to a woman who had been losing blood several hours. By touching he recognized the placenta inverted on the neck. Having introduced three fingers behind the symphysis, partly to detach the placenta, he ruptured the bag of waters, and slowly penetrated into the interior of the womb. The head was in the first position at the superior straight; he applied the forceps in this straight, which, on account of the space in which we manœuvre, is easier than generally supposed. The child was withdrawn alive, but half asphyxiated. While our cares were directed to its recovery, the sound of a liquid running on the floor apprized us that the mother was bleeding freely.

"Notwithstanding her delivery the blood continued to flow, and the patient's faintings continually increased; M. Pinel, after having in vain injected cold water, and squeezed several lemons in the cavity of the womb, resorted to compression of the aorta, which momentarily arrested the flooding; but as soon as they ceased applying the fingers the blood reappeared as abundantly as before. At length, an hour having elapsed, it seemed to run somewhat less. A pealed lemon was introduced anew, and the hemorrhage gradually stopped.

"These cases, those reported by the authors, sufficiently attest the utility of compression of the aorta. But this point established, it remains for us to consider the place to be chosen for the operation, the manner in which it is to be performed, and its duration.

"All the modifications proposed can be reduced to three procedures. Some, as Budiger, Eichelberg, carry the hand into the uterus; others, as Saxtorph, reach the aorta by acting on the womb through the walls of the abdomen; finally, the third party, whose method is generally followed now, compress the artery above the womb.

"The introduction of the hand into the uterus, and resting on its posterior region, has been rightly blamed. This plan indeed is bad, and of difficult application; it exposes the tissue of the organ to a kind of attrition; it is moreover imprac-

*Censeur Medical, Avril, 1834, p. 301.

ticable when the womb begins to react. However, it has many times succeeded. Eichelberg cites the case of a woman in whom compression thus effected lasted an hour; the moment it was stopped blood flowed.

"The physical condition of the female just delivered is favorable for compressing the aorta independently of the thinness of the walls, which, by the spreading out of the recti abdominis muscles, are reduced to that of the skin and two aponeurotic and serous membranes, which allow the aorta and vena cava to be almost directly touched; the intestines have, so to speak, chosen an abode in the lateral portions of the abdomen.

"The fundus of the womb also can easily be pushed into the region of the loins, or into the pelvis, whilst in the normal state, besides the thickness, somewhat considerable, of the walls, due to the accumulation of fatty cellular tissue, we are obliged to press on the intestines in order to reach the aorta, which makes the operation more difficult and painful.

"Compression through the abdominal parietes can be exerted with the thumb, with two or with four fingers, as preferred by MM. Baudeloque, Tréhan and Ulsamer. We have seen that Siebold had practised it with the closed hand applied a little to the left of the spine; this plan is more difficult in execution than the two preceding. M. Piedagnel employed the cubital margin of his hand. M. Pinel Grandchamp, advises us to press the artery moderately with the fingers; in bearing forcibly on the vessel the fingers grow numb and the operation cannot be continued long by the same person. This means, it appears to us, ought to be employed in preference to all others. To reach the aorta, the precaution must be taken to turn aside the intestines; the arterial pulsation indicates the presence of the vessel. It is then compressed longitudinally without involving the inferior vena cava in the manœuvre.

"The duration of the compression has been a matter of very diverse opinions. Some have limited it to five, six and seven minutes; others have prolonged it an hour or two. Eichelberg and M. Pinel Grandchamp, did not succeed in arresting the blood in less than an hour. M. Paul Dubois thinks we must continue the operation an hour or two, and then suspend it by degrees, assuring ourselves that the bleeding appears no more. The examples cited by us prove that simple compression suffices to check the hemorrhage, but we believe it better to associate with it the spurred rye, (ergot.)

"However it may be with the combination of these two means, we are not the less persuaded that compression of the aorta has been and will be of real service; moreover, M. Piedagnel appears to us to have done well in adding his observations to those of Blount,* of MM. Brossart,† Latour,‡ Lowenhardt,§ and Martins.||—*La Lancette Francaise, May* 12, 1840.

*Ingleby on Uterine Hemorrhagy, p. 249.
†Thesis, Strasbourg, Feb. 1830.
‡Revue Medicale, t. iii., p. 22, 1830.
§Revue Medicale, *id.* ||*Ibid.*

